# Genetic landscape of phenylketonuria in Brazil

**DOI:** 10.1186/s13023-026-04435-x

**Published:** 2026-07-09

**Authors:** Rafael Hencke Tresbach, João Braga de Abreu Neto, Fernanda Sperb-Ludwig, Marta Wey Vieira, Roseli Divino Costa, Pedro Eduardo Bonfim Freitas, Luiz Carlos Santana da Silva, Louise Lapagesse de Camargo Pinto, Marcial Francis Galera, Keyla Christy Christine Mendes Sampaio Cunha, Cezar Antonio Abreu de Souza, José Nélio Januário, Marcos José Burle Aguiar, Romina Soledad Heredia, Maria Teresa Alves da Silva Rosa, Monique Oliveira Poubel, François Maillot, Ida Vanessa Doederlein Schwartz

**Affiliations:** 1 BRAIN Laboratory (Basic Research and Advanced Investigations in Neurosciences), Experimental Research Center, Hospital de Clínicas de Porto Alegre, Porto Alegre, Brazil; 2 Brazilian National Institute on Rare Diseases, Porto Alegre, Brazil; 3 Post-Graduation Program in Genetics and Molecular Biology, Universidade Federal do Rio Grande do Sul, Porto Alegre, Brazil; 4 School of Medical and Health Sciences, Pontifícia Universidade Católica de São Paulo, São Paulo, Brazil; 5 Universidade Federal de Mato Grosso, Cuiabá, Brazil; 6 Inborn Errors of Metabolism Laboratory, Institute of Biological Sciences, Universidade Federal do Pará, Belém, Brazil; 7 Medical Genetics Service, Hospital Infantil Joana de Gusmão, Florianópolis, Brazil; 8 Department of Pediatrics, School of Medicine, Universidade Federal de Mato Grosso, Cuiabá, Brazil; 9 Center for Diagnostic Support Actions and Research (NUPAD), School of Medicine, Universidade Federal de Minas Gerais, Belo Horizonte, Brazil; 10 Newborn Screening Service of Brasilia, Brasília, Brazil; 11 Genetics Unit, Hospital de Apoio de Brasília, Brasília, Brazil; 12 CHRU et Université de Tours, INSERM 1253 “iBrain”, Tours, 37032 France; 13 Medical Genetics Service, Hospital de Clínicas de Porto Alegre, Porto Alegre, Brazil

**Keywords:** Phenylketonuria, Genotype, Population genetics

## Abstract

Phenylketonuria (PKU), one of the most common inherited metabolic disorders, is caused by biallelic loss-of-function variants in the *phenylalanine hydroxylase* (*PAH*) gene. More than 1000 pathogenic variants have been described in this gene. Although genotype-phenotype correlations are imperfect, *PAH* genotypes can influence treatment decision-making. To better characterize the genetic landscape of PKU in Brazil, we compiled a dataset of *PAH* genotypes from 742 patients by combining data from the literature (*n* = 486), public databases (*n* = 117), and direct physician reports (*n* = 139). Most patients were classified as having classical PKU (*n* = 455/742; 61.3%). The most prevalent variants were c.1162G > A (16.6%), c.1066-11G > A (13.9%), and c.782G > A (11.8%), which are also the predominant ones in Portugal, Spain, and Latin America. This study confirms the allelic heterogeneity of PKU in the Brazilian population and provide relevant insights to support public health policies for this disorder.

**Supplementary Information:**

The online version contains supplementary material available at 10.1186/s13023-026-04435-x.

## Background

Phenylketonuria (PKU, OMIM #61600), one of the most common inherited metabolic disorders, is caused by biallelic loss-of-function variants in the *phenylalanine hydroxylase* (*PAH*) gene. This gene encodes the liver enzyme PAH (EC 1.14.16.1), responsible for hydroxylating phenylalanine (Phe) to tyrosine (Tyr). PAH activity requires tetrahydrobiopterin as a co-substrate, as well as iron and molecular oxygen as cofactors. DNAJC12 protein also plays a role as a co-chaperone in the Phe-to-Tyr conversion [1], while the long noncoding RNA *HULC* has been implicated in the regulation of PAH activity [2].

The *PAH* gene is located on chromosome 12 and comprises 13 exons and 12 introns, encoding a tetrameric protein with 452 amino acid residues [3]. To date, 3,457 variants have been described in PAHvdb (http://www.biopku.org/*)*, and 1,221 pathogenic variants have been reported in the Human Gene Mutation Database [4], highlighting the allelic heterogeneity of the *PAH* gene [5].

The worldwide prevalence of PKU is estimated at 1:23,930 live births [6]. In Latin America, reported incidence ranges from 1:15,000 to 1:50,000 births [7, 8], while in Brazil it is estimated at 1:25,000 births [6, 9]. In the United States, PKU prevalence ranges from 1:9,000 to 1:33,000 [10], while in Europe the mean prevalence is approximately 1:10,000 births, with inter-country variability [5, 6].

Untreated PKU leads to irreversible neurological damage, impaired cognitive development, and seizures [5]. Early diagnosis is essential to avoid these consequences. In Brazil, newborn screening for PKU began in 1976 by isolated initiatives and, following improvements in public health policies over decades, mainly after 2001, has become widely implemented nationwide [10]. However, disparities in screening coverage persist across Brazilian states - while the public national coverage increased from about 55% in 2000 to 82.7% newborns (NB) in 2024, the coverage in the states ranged from 58% in the northern state of Amapá to 96.94% in Alagoas (Northeast region) in 2024 [11, 12].

Population genetic studies of inherited metabolic disorders are of major importance in public health policy, as they support diagnostic strategies, inform genetic counseling, clarify disease distribution across the country, and contribute to treatment decision-making. In this study, we compiled available genotype data from Brazilian patients with PKU to provide a comprehensive overview of the molecular landscape of the disease in Brazil.

## Methods

This study was approved by the local Institutional Review Board (CAAE no. 51058121.8.0000.5327). Genotype data were obtained from multiple sources, including published literature, physician reports, and the BioPKU database.

### Literature search

PubMed and SciELO databases were searched for studies published between August 1995 and November 2025 using the following search terms: “phenylketonuria AND Brazil”, “phenylketonuria AND Brazilian”, “fenilcetonúria AND Brasil”, “fenilcetonúria AND brasileiro”. An additional search for “fenilcetonúria” was performed in the Brazilian thesis and dissertation database Sucupira (https://sucupira.capes.gov.br/) to capture genotype data not reported in peer-reviewed articles.

### Databases

BioPKU (http://www.biopku.org/) is an international database that compiles clinical, genotypic, and phenotypic information on patients with PKU around the world [13]. Professor Nenad Blau, curator of the database, kindly shared genotypes and PKU types compiled from Brazilian patients registered in BioPKU up to October 2019.

### Physician reports

A network of Brazilian physicians from the *Sociedade Brasileira de Genética Médica e Genômica* (Brazilian Society of Medical Genetics and Genomics) and the *Sociedade Brasileira de Triagem Neonatal e Erros Inatos do Metabolismo* (Brazilian Society of Newborn Screening and Inborn Errors of Metabolism) were invited to collaborate on this project. Healthcare professionals submitted patient data through a standardized electronic form, including anonymized identification of the patient, gender, PKU type/severity, results of tetrahydrobiopterin (BH_4_) responsiveness testing, patient genotype, methods used for genotyping, and references to published articles when patient data were published. The origin of the patient was considered as the state/Brazilian region where the patient was born in.

PKU type/severity was classified according to daily Phe tolerance: pretreatment blood Phe levels > 1200 µMol/L corresponded to classical PKU (cPKU); levels between 600 and 1200 µMol/L, to mild PKU (mPKU); and levels between 120 and 600 µMol/L, to non-PKU hyperphenylalaninemia (HPA) [14].

### Data analysis

All data were compiled into a large dataset, including data source, phenotype classification, and available clinical information. Allelic phenotype values (APVs) were retrieved from BioPKU when available [13].

To investigate the effects of allele frequency on the variants identified in our cohort, population allele frequency data were incorporated from the global human Genome Aggregation Database (gnomAD), including 730,947 exomes [15] and 76,215 genomes, as well as from the Online Archive of Brazilian Mutations (ABraOM), containing 1171 admixed older individuals from São Paulo, Brazil [16].

Populational data of each geographic region of the country was collected at last report of the Brazilian Institute of Geography and Statistics (IBGE) [17].

All data were analyzed using the Google Sheets platform. Data entries were manually curated to standardize genetic variant annotations according to the current Human Genome Variation Society (HGVS) nomenclature guidelines [18].

## Results

Genotype data from 742 Brazilian patients with PKU were compiled (117 from the BioPKU database, 486 identified through the literature review, and 139 unpublished cases reported by physicians in our collaborative network) (Supplementary Table S1). Although a total of 209 cases were directly reported to us by physicians, 70 of these cases had already been described and were therefore included through the literature review. Despite the relatively large number of publications on PKU in Brazil, only a small subset reported patient genotypes. Our search identified 12 peer-reviewed articles and 12 academic theses containing genotype data (Table 1). Potential duplicate records were carefully reviewed and excluded or merged when appropriate.

**Table 1:** Literature review of articles and academic theses reporting genetic information on patients with phenylketonuria (PKU) in Brazil

Authors and year	Patients included in the review (*n*)	Brazilian region of origin	Most common variant found	References
Acosta, AX et al. (2000); Acosta, AX (2001)*	0	Southeast	c.1066-11G > A	[19, 20]
Steiner CE, Acosta AX, et al. (2007)	3	Southeast	c.1066-11G > A	[21]
Magalhães, M de C (2003); Santos LL (2004); Santos LL et al. (2006)*	0	Southeast	c.1162G > A	[22–24]
Santos, LL (2007); Santos LL, et al. (2008)	54	Southeast	c.1162G > A	[25, 26]
Pollice, EL (2008)	14	Southeast	c.194T > C	[27]
Vieira Neto, E. (2018); Vieira Neto, E et al.(2018); Vieira Neto, E et al. (2019)	102	Southeast	c.1162G > A	[28–30]
Amorim, T. (2010)	104	Northeast	c.1066-11G > A	[31]
de Santana Santos, E et al. (2012)	15	Northeast	c.1066-11G > A	[32]
Silva, CAN (2018)	106	Northeast	c.1066-11G > A	[33]
Bonfim-Freitas, PE (2006); Bonfim-Freitas PE, et al. (2023)	22	North	c.1066-11G > A	[34, 35]
Costa, RD (2017); Costa, RD; et al. (2020)	9	Central-West	c.1162G > A	[36, 37]
Santana da Silva, LC (2000); Santana da Silva LC, et al.(2003)	18	South	c.194T > C	[38, 39]
Tresbach, RH, et al. (2020); Tresbach, RH (2021)	34	South /Central-West	c.1315 + 1G > A	[40, 41]
Nunes, AJ, et al. (2025)	5	South	c. 1222 C > T	[42]
Total patients from the literature review (n)	486			
Total patients from BioPKU (n)	117			
Total patients reported directly by physicians (n)	139			
Total patients included in the study (n)	742			

Note: Each row corresponds to a patient group described in the publication(s) referenced in the same row. *Patients were not included in the present study because they had already been reported in the BioPKU database

**Fig. 1:**
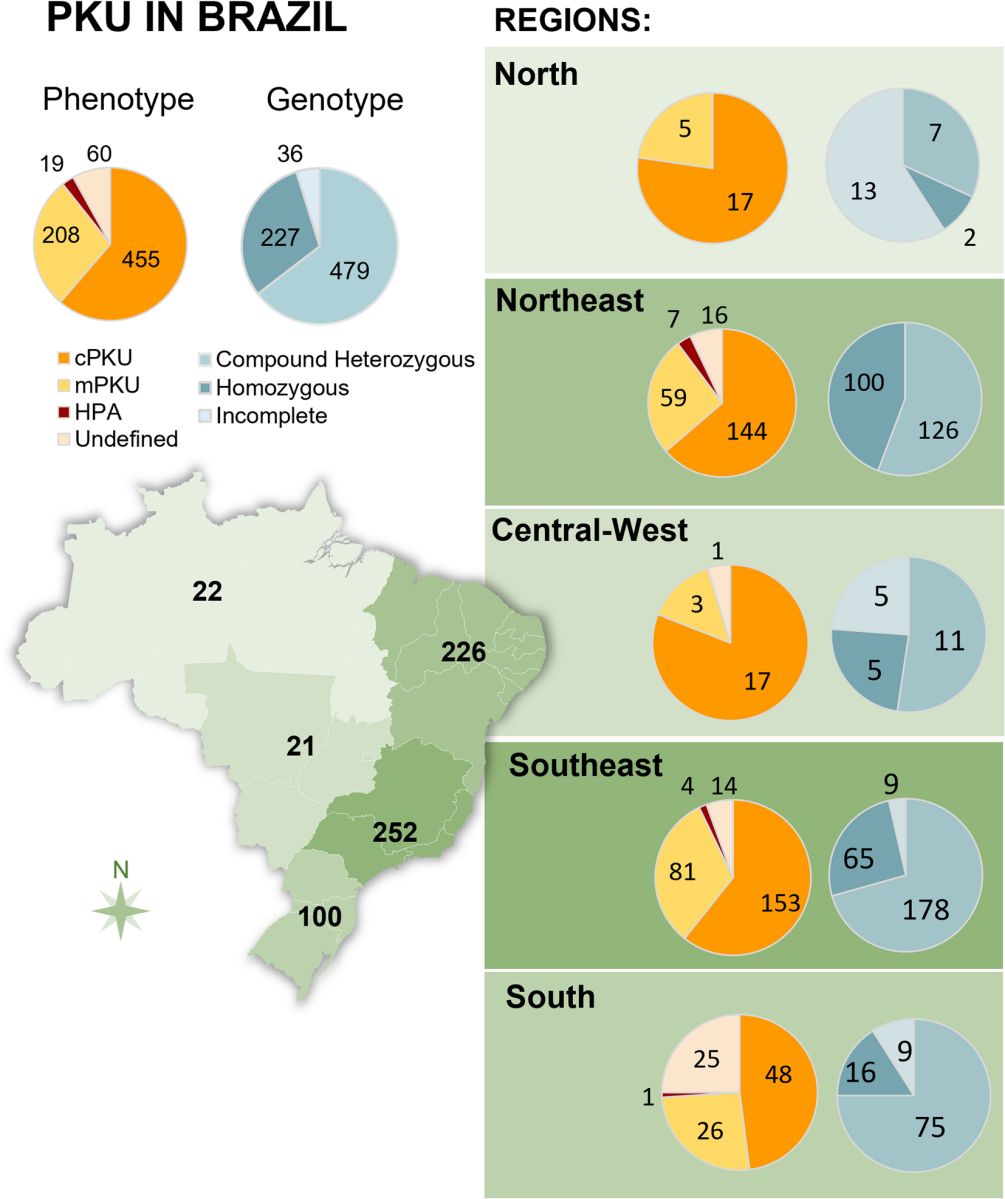
Phenotypic and genotypic classification of the 742 individuals included in the study, distributed across the five geographic regions of Brazil (North, Northeast, Central-West, Southeast, and South). Geographic information was unavailable for 121 patients; therefore, 621 individuals are represented on the map. PKU: phenylketonuria; cPKU: classical PKU; mPKU: mild PKU; HPA: non-PKU hyperphenylalaninemia; Undefined: classification unavailable or not reported

The final dataset consisted of 227 homozygous genotypes, 479 compound heterozygotes, and 36 incomplete genotypes (Fig. 1). Most incomplete genotypes resulted from limited genotyping methods used in some centers, such as targeted variant analysis. Most genotypes found were obtained by these targeted methods (*n* = 363), or did not disclose the implemented method (*n* = 194), while more recent genotypes were obtained by Sanger sequencing (*n* = 130), or by massive parallel sequencing (*n* = 55). The gender of the patients was disclosed by the source in only 28.4% of the cases (M = 107, F = 104, NA = 531) (Supplementary table S1).

### Origin (supplementary table S1 and Table 3)

This study included patients distributed across all five geographic regions of Brazil, but for 121 individuals the geographic information was unavailable (Fig. 1; Table 2 – for the full version, see Supplementary Table S2).

**Table 2:** The 15 most common *PAH* genotypes reported in Brazilian patients with phenylketonuria (PKU), stratified by geographic region and PKU type, with their respective frequencies in our dataset. The complete list of the 219 identified genotypes is provided in the supplementary materials (Table S2)

*N*	Genotype		Brazil	Region						PKU type			
	Allele 1	Allele 2	Total	South	Southeast	Central-West	Northeast	North	Not disclosed	cPKU	mPKU	HPA	NA
			*N* = 742 (%)	*N* = 100 (%)	*N* = 252 (%)	*N* = 21 (%)	*N* = 226 (%)	*N* = 22 (%)	*N* = 121 (%)	*N* = 455	*N* = 208	*N* = 19	*N* = 60
1	c.1162G > A	c.1162G > A	56 (7.5)	3 (3)	14 (5.6)	2 (9.5)	24 (10.6)	0 (0)	13 (10.7)	46/56 (82.1%)	8/56 (14.3%)	0/56 (0%)	2/56 (3.6%)
2	c.1066-11G > A	c.1066-11G > A	42 (5.7)	1 (1)	8 (3.2)	1 (4.8)	24 (10.6)	2 (9.1)	6 (5)	35/42 (83.3%)	7/42 (16.7%)	0/42 (0%)	0/42 (0%)
3	c.782G > A	c.782G > A	36 (4.9)	1 (1)	14 (5.6)	0 (0)	13 (5.8)	0 (0)	8 (6.6)	15/36 (41.7%)	19/36 (52.8%)	0/36 (0%)	2/36 (5.5%)
4	c.1066-11G > A	c.1162G > A	29 (3.9)	0 (0)	9 (3.6)	1 (4.8)	19 (8.4)	0 (0)	0 (0)	18/29 (62.1%)	10/29 (34.5%)	0/29 (0%)	1/29 (3.4%)
5	c.754 C > T	c.754 C > T	26 (3.5)	0 (0)	2 (0.8)	0 (0)	24 (10.6)	0 (0)	0 (0)	20/26 (76.9%)	5/26 (19.3%)	0/26 (0%)	1/26 (3.8%)
6	c.194T > C	c.194T > C	23 (3.1)	2 (2)	7 (2.8)	0 (0)	11 (4.9)	0 (0)	3 (2.5)	16/23 (69.6%)	3/23 (13%)	0/23 (0%)	4/23 (17.4%)
7	c.194T > C	c.1066-11G > A	21 (2.8)	0 (0)	1 (0.4)	0 (0)	20 (8.8)	0 (0)	0 (0)	14/21 (66.7%)	5/21 (23.8%)	0/21 (0%)	2/21 (9.5%)
8	c.782G > A	c.1162G > A	20 (2.7)	0 (0)	9 (3.6)	1 (4.8)	10 (4.4)	0 (0)	0 (0)	11/20 (55%)	9/20 (45%)	0/20 (0%)	0/20 (0%)
9	c.194T > C	c.1162G > A	18 (2.4)	0 (0)	8 (3.2)	0 (0)	9 (4)	1 (4.5)	0 (0)	12/18 (66.7%)	4/18 (22.2%)	1/18 (5.5%)	1/18 (5.6%)
10	c.782G > A	c.1066-11G > A	15 (2)	0 (0)	6 (2.4)	2 (9.5)	7 (3.1)	0 (0)	0 (0)	10/15 (66.7%)	4/15 (26.6%)	0/15 (0%)	1/15 (6.7%)
11	c.754 C > T	c.1162G > A	12 (1.6)	0 (0)	6 (2.4)	0 (0)	6 (2.7)	0 (0)	0 (0)	9/12 (75%)	1/12 (8.3%)	1/12 (8.3%)	1/12 (8.3%)
12	c.754 C > T	c.782G > A	11 (1.5)	1 (1)	6 (2.4)	0 (0)	4 (1.8)	0 (0)	0 (0)	7/11 (63.6%)	4/11 (36.4%)	0/11 (0%)	0/11 (0%)
13	c.782G > A	c.1315 + 1G > A	10 (1.3)	4 (4)	0 (0)	1 (4.8)	0 (0)	0 (0)	5 (4.1)	8/10 (80%)	2/10 (20%)	0/10 (0%)	0/10 (0%)
14	c.194T > C	c.754 C > T	9 (1.2)	0 (0)	0 (0)	0 (0)	9 (4)	0 (0)	0 (0)	6/9 (66.7%)	3/9 (33.3%)	0/9 (0%)	0/9 (0%)
15	c.473G > A	c.1162G > A	9 (1.2)	3 (3)	1 (0.4)	0 (0)	0 (0)	0 (0)	5 (4.1)	4/9 (44.4%)	5/9 (55.6%)	0/9 (0%)	0/9 (0%)

Note: NA: PKU type classification inconclusive or not available

**Table 3:** Geographical origin (Brazilian regions): comparison between PKU patients and the total population

	Brazil	Region					
	Total (*n*)	Southeast (*n*)	Northeast (*n*)	South(*n*)	North (*n*)	Central-West (*n*)	Not Available
**a. Populational data**							
Population (IBGE 2025)[17]	213,421,037 (100%)	88,825,643 (41.6%)	57,244,485 (26.8%)	31,310,809 (14.7%)	18,801,282 (8.8%)	17,238,818 (8.1%)	
PKU cases 2008–2021 [11]	7,615 (100%)	4,038 (53.0%)	1,508 (19.8%)	946 (12.4%)	541 (7.1%)	582 (7.7%)	
**b. Our cohort**							
Genotyped patients (n)	742 (100%)	252 (34.0%)	226 (30.4%)	100 (13.5%)	22 (3.0%)	21 (2.8%)	121 (16.3%)
Different variants reported (n)	84	47	15	43	17	11	51
Five most frequent variants	c.1162G > A (16.6%)	c.1162G > A (16.9%)	c.1066-11G > A (25%)	c.194T > C (12%)	c.1066-11G > A (11.4%)	c.1066-11G > A (19%)	c.1162G > A (15.7%)
	c.1066-11G > A (13.9%)	c.782G > A (14.5%)	c.1162G > A (21.5%)	c.1222 C > T (11%)	c.782G > A (11.4%)	c.782G > A (19%)	c.1066-11G > A (6.6%)
	c.782G > A (11.8%)	c.1066-11G > A (11.9%)	c.754 C > T (17.3%)	c.1162G > A (8%)	c.1162G > A (9.1%)	c.1162G > A (16.7%)	c.782G > A (10.7%)
	c.194T > C (9.5%)	c.194T > C (6.7%)	c.194T > C (15%)	c.1315 + 1G > A (7.5%)	c.194T > C (6.8%)	c.184delC (9.5%)	c.194T > C (5%)
	c.754 C > T (8.2%)	c.754 C > T (5.6%)	c.782G > A (11.5%)	c.782G > A (5.5%)	c.745 C > T (4.5%)	c.473G > A (4.8%)	c.754 C > T (2.5%)

Most included studies reported data on patients from only one state or region of the country. From the Southeast, data were obtained for 102 patients from Rio de Janeiro [28], 17 from São Paulo [21, 27], and 54 from Minas Gerais [25]. From the North, 22 patients were reported from Pará [35]. From the Northeast, data included 15 patients from Alagoas [32], 45 patients from Bahia [33], and 104 patients from five different states reported in a thesis by T. Amorim (2010) [31]. From the Central-West, 9 patients were from Mato Grosso [37] and 11 were from the Federal District [40]. From the South, 46 patients were reported from Rio Grande do Sul and Santa Catarina [39, 40, 42]. Our previous study [40] was unique in including patients from both the South and Central-West regions.

Table 3 shows a comparison between the geographical distribution of included patients and the Brazilian population. Patients from the North (3%) and Central-West (2.8%) regions seem to be underrepresented in our sample, since they should correspond to about 8% of the sample each if we assume the prevalence of PKU is equal among all Brazilian regions.

### PKU type & BH4 responsiveness

Most patients were classified as having cPKU (*n* = 455; 61.3%) or mPKU (*n* = 208; 28%), while 19 (2.6%) were classified as having non-PKU HPA and 60 (8.1%) had undefined or unavailable classification.

The results of BH_4_ responsiveness testing were unavailable for most patients in our cohort (*n* = 687; 92.6%). Among the 55 patients (7.4%) with available results, 36 (4.9%) were classified as BH_4_ non-responders, 1 (0.1%) as slow responder, and 18 (2.4%) as responders (Table 4).

**Table 4:** Results of tetrahydrobiopterin (BH4) responsiveness testing in patients included in the dataset

BH_4_ responsiveness test	Total of patients
Responders	18 (2.4%)
Slow responders	1 (0.1%)
Non-responders	36 (4.9%)
Test not performed	687 (92.6%)

### Genotypes & variants

A total of 219 distinct genotypes were identified. The three most frequent were homozygous for the variants c.1162G > A(;)1162G > A (56 patients), c.1066-11G > A(;)1066-11G > A (42 patients), and c.782G > A(;)782G > A (36 patients). In contrast, 104 genotypes were reported in only one patient, and 45 genotypes were reported in two patients (Supplementary Table S2).

Eighty-four unique variants were identified (Table 5). The most frequent variant found in Brazil was c.1162G > A (p.Val388Met), followed by c.1066-11G > A (p.Gln355_Tyr356insGlyLeuGln) and c.782G > A (p.Arg261Gln). These three variants were among the most common across all regions of the country, although in different order. Interestingly, c.194T > C (p.Ile65Thr) was the most frequent variant in patients from the South region (12%) but was not detected among the 21 patients from the Central-West region. Conversely, c.184delC (p.Leu62Ter) accounted for 9.5% of variants in the Central-West but was not reported in other regions (Table 3). The South region presented the most diverse variant profile: it was the only one in which the c.1162G > A (p.Val388Met) variant was not the most frequent, and the only one in which the c.1222 C > T (p.Arg408Trp) and c.1315 + 1G > A variants were among the five most frequent (Table 3).

**Table 5:** Pathogenic *PAH* variants identified in Brazilian patients with phenylketonuria (PKU) and their respective allele frequencies in the study cohort and in the gnomAD and ABraOM databases (when available), as well as variant location in the gene (intron [I], exon [E], or untranslated region [UTR] region) and phenotype classification according to the BioPKU database

No.	Variant				Study cohort (AF)	gnomAD (AF)	ABraOM (AF)	BioPKU	
	Nucleotide change	Protein change	Location	Total alleles				APV*	Phenotype
1	c.1162G > A	p.Val388Met	E11	247	0.16644	0.00004	0.00043	2.1	cPKU
2	c.1066-11G > A	p.Gln355_Tyr356insGlyLeuGln	I10	207	0.13949	0.00032	0.00085	0	cPKU
3	c.782G > A	p.Arg261Gln	E7	175	0.11792	0.00023	0.00128	1.5	cPKU
4	c.194T > C	p.Ile65Thr	E3	141	0.09501	0.00060	NA	1.5	cPKU
5	c.754 C > T	p.Arg252Trp	E7	122	0.08221	0.00004	NA	0	cPKU
6	c.1222 C > T	p.Arg408Trp	E12	51	0.03437	0.00132	0.00043	0	cPKU
7	c.168 + 5G > C	NA	I2	44	0.02965	0.00002	NA	0	cPKU
8	c.1315 + 1G > A	NA	I12	44	0.02965	0.00094	0.00043	0	cPKU
9	c.473G > A	p.Arg158Gln	E5	35	0.02358	0.00014	0.00085	0	cPKU
10	c.1042 C > G	p.Leu348Val	E10	31	0.02089	0.00021	0.00043	1.4	cPKU
11	c.1169 A > G	p.Glu390Gly	E11	28	0.01887	0.00010	NA	6.8	mPKU/HPA
12	c.745 C > T	p.Leu249Phe	E7	26	0.01752	0.00007	NA	2.5	cPKU
13	c.842 C > T	p.Pro281Leu	E7	23	0.01550	0.00012	NA	0	cPKU
14	c.168 + 5G > A	NA	I2	19	0.01280	0.00000	NA	0	cPKU
15	c.781 C > T	p.Arg261Ter	E7	19	0.01280	0.00003	0.00043	0	cPKU
16	c.1045T > C	p.Ser349Pro	E10	14	0.00943	0.00013	NA	0	cPKU
17	c.1241 A > G	p.Tyr414Cys	E12	13	0.00876	0.00035	NA	5.1	mPKU
18	c.250G > T	p.Asp84Tyr	E3	12	0.00809	0.00000	NA	5	mPKU
19	c.809G > A	p.Arg270Lys	E7	11	0.00741	0.00001	NA	0	cPKU
20	c.1055delG	p.Gly352ValfsTer48	E10	11	0.00741	NA	NA	0	cPKU
21	c.967_969del	p.Thr323del	E9	9	0.00606	0.00000	NA	0	cPKU
22	c.184delC	p.Leu62Ter	E3	8	0.00539	NA	NA	0	cPKU
23	c.526 C > T	p.Arg176Ter	E6	8	0.00539	0.00001	0.00043	0	cPKU
24	c.842 + 1G > A	NA	I7	7	0.00472	0.00006	NA	0	cPKU
25	c.472 C > T	p.Arg158Trp	E5	7	0.00472	0.00002	NA	0	cPKU
26	c.1243G > A	p.Asp415Asn	E12	7	0.00472	0.00007	0.00043	9.8	HPA
27	c.728G > A	p.Arg243Gln	E7	6	0.00404	0.00004	NA	0.4	cPKU
28	c.441 + 5G > T	NA	I4	6	0.00404	0.00002	NA	0	cPKU
29	c.165delT	p.Phe55LeufsTer6	E2	6	0.00404	NA	NA	0	cPKU
30	c.838G > A	p.Glu280Lys	E7	5	0.00337	0.00016	NA	0	cPKU
31	c.1199 + 17G > A	NA	I11	5	0.00337	0.00000	0.00043	8.3	HPA
32	c.527G > T	p.Arg176Leu	E6	5	0.00337	0.00005	0.00086	9.8	HPA
33	c.932T > C	p.Leu311Pro	E9	4	0.00270	0.00001	NA	0	cPKU
34	c.934G > T	p.Gly312Cys	E9	4	0.00270	NA	NA	5	mPKU
35	c.712 A > C	p.Thr238Pro	E7	4	0.00270	0.00000	NA	0	cPKU
36	c.442–5 C > G	NA	E2	4	0.00270	0.00001	NA	6.1	mPKU
37	c.926 C > T	p.Ala309Val	E9	4	0.00270	0.00002	NA	3.3	mPKU
38	c.722G > A	p.Arg241His	E7	3	0.00202	0.00009	NA	5	mPKU
39	c.1208 C > T	p.Ala403Val	E12	3	0.00202	0.00049	0.00043	9.7	HPA
40	c.1223G > A	p.Arg408Gln	E12	3	0.00202	0.00008	NA	4.7	mPKU
41	c.136G > A	p.Gly46Ser	E2	3	0.00202	0.00004	NA	1.6	cPKU
42	c.442-?_509+?del	NA	E5	3	0.00202	NA	NA	0	cPKU
43	c.442_509del168	NA	E5	3	0.00202	NA	NA	0	cPKU
44	c.814G > T	p.Gly272Ter	E7	3	0.00202	0.00005	NA	0	cPKU
45	c.143T > C	p.Leu48Ser	E2	3	0.00202	0.00009	NA	1.9	cPKU
46	c.721 C > T	p.Arg241Cys	E7	3	0.00202	0.00005	NA	5.1	mPKU
47	c.524 C > G	p.Pro175Arg	E6	2	0.00135	0.00000	NA	NA	NA
48	c.724 C > T	p.Leu242Phe	E7	2	0.00135	0.00000	NA	0	cPKU
49	c.43_44insCT	p.Leu15ProfsTer24	E1	2	0.00135	NA	NA	0	cPKU
50	c.116_118del	p.Phe39del	E2	2	0.00135	0.00001	NA	0.8	cPKU
51	c.561G > C	p.Trp187Cys	E6	2	0.00135	NA	NA	NA	NA
52	c.498 C > A	p.Tyr166Ter	E5	2	0.00135	NA	NA	0	cPKU
53	c.503del	p.Tyr168SerfsTer27	E5	2	0.00135	0.00000	NA	0	cPKU
54	c.204 A > T	p.Arg68Ser	E3	2	0.00135	0.00007	0.00043	5.4	mPKU
55	c.1024delG	p.Ala342HisfsTer58	E10	2	0.00135	NA	NA	0	cPKU
56	c.833 C > T	p.Thr278Ile	E7	2	0.00135	0.00000	NA	0	cPKU
57	c.283 A > T	p.Ile95Phe	E3	1	0.00067	0.00001	0.00043	5	mPKU
58	c.898G > T	p.Ala300Ser	E8	2	0.00135	0.00040	0.00043	9.7	HPA
59	c.764T > C	p.Leu255Ser	E7	1	0.00067	NA	NA	0	cPKU
60	c.1065 + 1G > A	NA	I10	1	0.00067	0.00000	NA	0	cPKU
61	c.561G > A	p.Trp187Ter	E6	1	0.00067	0.00002	NA	0	cPKU
62	c.782G > C	p.Arg261Pro	E7	1	0.00067	0.00000	0.00128	2.6	cPKU/mPKU
63	c.169-13T > G	p.Glu57_Lys452delinsSerLauPhe	I2	1	0.00067	0.00000	NA	0	cPKU
64	c.163_165del	p.Phe55del	E2	1	0.00067	NA	NA	0	cPKU
65	c.994G > A	p.Gly332Arg	E10	1	0.00067	0.00000	NA	NA	NA
66	c.719T > C	p.Phe240Ser	E7	1	0.00067	NA	NA	NA	NA
67	c.359G > A	p.Trp120Ter	E4	1	0.00067	NA	NA	0	cPKU
68	c.614 A > C	p.Glu205Ala	E6	1	0.00067	NA	NA	NA	NA
69	c.799 C > T	p.Gln267Ter	E7	1	0.00067	NA	NA	0	cPKU
70	c.712 A > G	p.Thr238Ala	E7	1	0.00067	NA	NA	5	mPKU
71	c.441 + 4 A > G	NA	I4	1	0.00067	NA	NA	0	cPKU
72	c.960G > C	p.Lys320Asn	E9	1	0.00067	0.00000	NA	4.5	mPKU
73	c.331 C > T	p.Arg111Ter	E3	1	0.00067	0.00006	NA	0	cPKU
74	c.734T > C	p.Val245Ala	E7	1	0.00067	0.00054	NA	9.9	HPA
75	c.205 C > T	p.Pro69Ser	E3	1	0.00067	NA	NA	NA	NA
76	c.727 C > T	p.Arg243Ter	E7	1	0.00067	0.00010	0.00043	0	cPKU
77	c.168 + 5G > T	NA	I2	1	0.00067	NA	NA	0	cPKU
78	c.*19G > T	NA	UTR 3’	1	0.00067	0.00129	NA	NA	NA
79	c.618 C > A	p.Tyr206Ter	E6	1	0.00067	NA	NA	0	cPKU
80	c.842 + 1G > T	NA	I7	1	0.00067	NA	NA	0	cPKU
81	c.1232 C > G	p.Ser411Ter	E12	1	0.00067	NA	NA	0	cPKU
82	c.1069T > G	p.Cys357Gly	E11	1	0.00067	0.00000	NA	0	cPKU
83	c.649T > C	p.Cys217Arg	E6	1	0.00067	0.00000	NA	10	HPA
84	c.1229T > G	p.Phe410Cys	E12	1	0.00067	NA	NA	NA	NA

**Notes**: *Gene location: intron (I) or exon (E); **Allelic phenotype values (APV) and associated phenotype classification as defined in the BioPKU database. NA: not available; cPKU: classical PKU; mPKU: mild PKU; HPA: non-PKU hyperphenylalaninemia; AF: allele frequency; gnomAD: Genome Aggregation Database; ABraOM: Online Archive of Brazilian Mutations

**Fig. 2:**
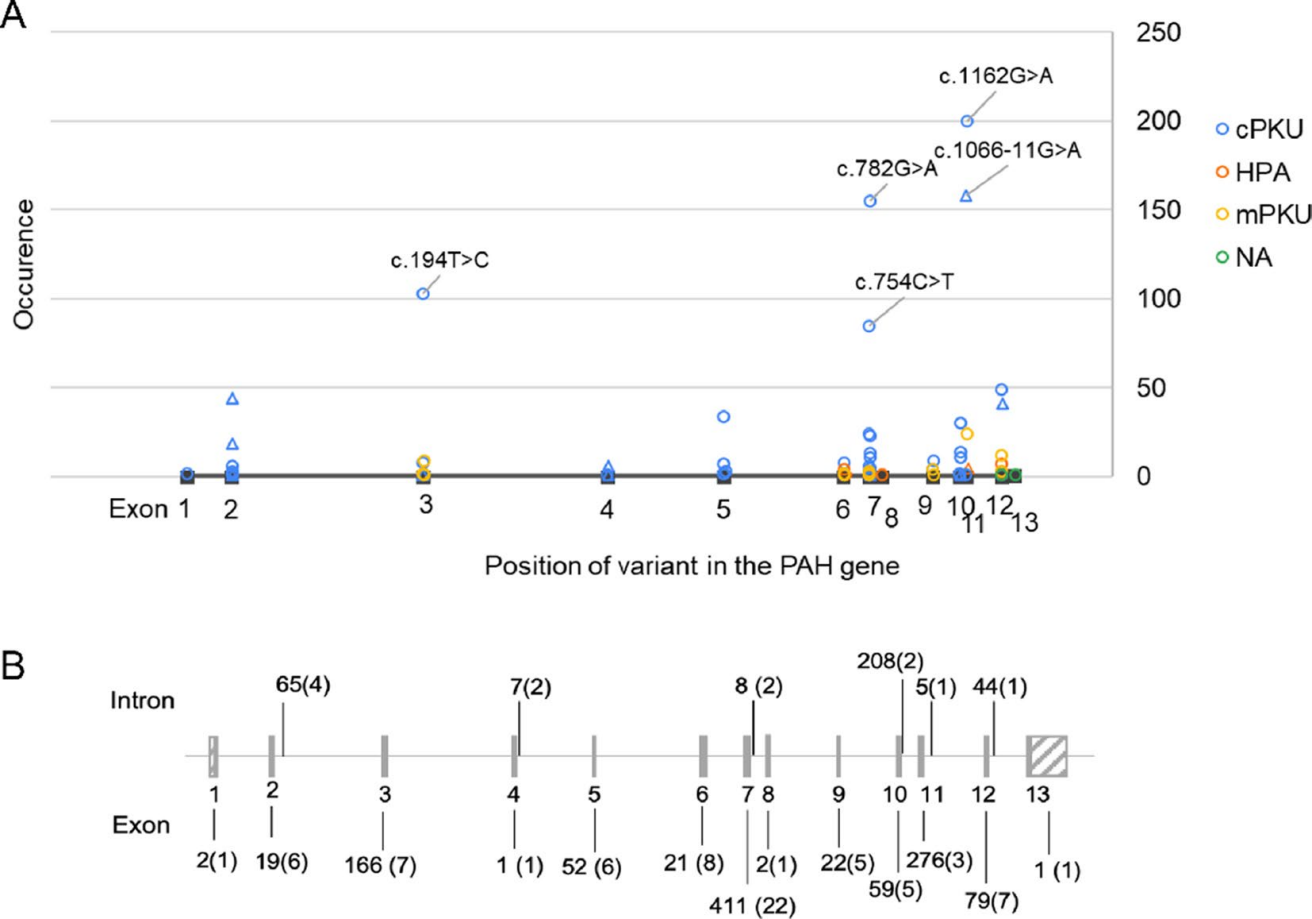
**(A)** Distribution of the 84 distinct variants from a total of 1448 found in the study cohort along the *PAH* gene sequence, based on chromosomal position (Ghr38:Chr12:102,930,000-102,830,000). The five most frequent variants are described; the complete list of variants is provided in Table 3. Point colors denote phenotype classification according to the BioPKU database: classical PKU (cPKU, blue), mild PKU (mPKU, yellow), non-PKU hyperphenylalaninemia (HPA, orange), and variants with unavailable classification (NA, green). Circles represent variants located in exonic regions, while triangles indicate variants located in intronic regions. Exons are represented by black boxes along the graph axis. **(B)** Relative distribution of the 1448 variants in the *PAH* gene, showing the number of variants at each position (values in parentheses indicate the number of distinct variants per region)

In our dataset, 71 different variants were located in exonic regions of the *PAH* gene, while 12 different intronic variants were identified, all located near exon-intron boundaries (Fig. 2). Although pathogenic variants were found in every exon, their distribution was not uniform throughout the gene, with the majority (73.3%) located in intron 11 and exons 3, 7, and 11 (Fig. 2).

## Discussion

To our knowledge, this study represents the most comprehensive compilation of genetic data from Brazilian patients with PKU, encompassing information from 742 individuals. A previous study compiling data from Brazilian patients was able to obtain information from 219 individuals, only including cases deposited in the BioPKU database [6].

The Brazilian population is characterized by substantial genetic heterogeneity resulting from complex colonization and immigration processes, with contributions primarily from European (EUR), African (AFR), and Native American (AME) ancestries. The relative proportions of these genetic backgrounds vary across the five regions of the country (North, Northeast, Central-West, Southeast and South): in the South, the prevalence is 80.0% EUR, 10.9% AFR, and 8.9% AME; in the Southeast, 69.1% EUR, 21.7% AFR, and 8.2% AME; in the Central-West, 63.4% EUR, 24.4% AFR, and 13.2% AME; in the Northeast, 55.3% EUR, 28.8% AFR, and 15.8% AME; and in the North, 52.9% EUR, 19.9% AFR, and 27.3% AME [43]. This information is important for understanding of the genetic findings we obtained.

### Genetic findings

The four most common variants found in this study, c.1162G > A (p.Val388Met, 16.6%), c.1066-11G > A (p.Gln355_Tyr356insGlyLeuGln, 13.9%), c.782G > A (p.Arg261Gln, 11.8%) and c.194T > C (p.Ile65Thr, 9.5%), are also the most frequent pathogenic variants in PKU patients from Portugal, the country most involved in the European colonization of Brazil, although at inverted frequencies in the Portuguese population (8.3%, 8.3%, 13.4%, and 14.1%, respectively) [44]. The most frequent variant identified in the Brazilian population, c.1162G > A (p.Val388Met), has also been a common pathogenic variant in Latin America and Spain [6, 45]. In contrast, c.1222 C > T (p.Arg408Trp), described as the most prevalent PKU-associated variant worldwide [6], ranked only sixth in variant distribution in our cohort of Brazilian patients. However, it was the second most prevalent variant in the South region of the country, the region with the greatest European ancestry, especially descendants of Germans and Italians.

Consistent with previous studies [28], considerable phenotypic variability was observed among individuals sharing identical genotypes. Of the 219 distinct genotypes identified, 48 were associated with two different PKU classes – including the prevalent c.1162G > A;c.1162G > A (46 cPKU, 8 mPKU) and c.1066-11G > A;c.1066-11G > A (35 cPKU, 7 mPKU) – and three were associated with three classes c.194T > C;c.1162G > A, c.754 C > T;c.1162G > A, and c.745 C > T;c.1162G > A. This variability may be partly explained by differences in phenotypic classification methods, particularly the timing of pretreatment Phe measurements, which can vary across centers and increase daily with postnatal age in untreated infants [28, 46]. Moreover, the multicenter nature of this study likely contributed additional variability in sample collection timing.

Data on BH_4_ responsiveness were available for only a small subset of patients included in this study (*n* = 55; 7.4%), reflecting the fact that BH_4_ responsiveness testing is not routinely performed in Brazil. This limitation is partly due to the high cost of sapropterin dihydrochloride [5, 47], the drug used to supplement BH_4_ in responders, and its non-inclusion in the main treatment options for patients with PKU by the corresponding committee which evaluates the incorporation of treatments in the Brazilian public health system [48]. Nevertheless, *PAH* genotyping remains a valuable tool for identifying potentially responsive patients and for minimizing misclassifications of BH_4_ responsiveness [28].

### Sample representativeness

A recent study identified 7,615 patients with a confirmed diagnosis of PKU registered in the Brazilian public health system (SUS) [11], suggesting that our sample may correspond to approximately 10% of living PKU patients in Brazil. Some factors may bias the representativeness of our sample, such as the availability of genetic testing and the coverage of neonatal screening.

Unfortunately, the genetic testing has not yet been included in the “standard of care” for affected individuals in the country, since the test is not fully covered by SUS nor is it necessary for reimbursement of the metabolic formula by the health system. We emphasize that most Brazilian PKU patients are monitored at Neonatal Screening Reference Centers or Rare Disease Reference Centers, distributed throughout the country and maintained by the SUS. Analysis of the source data included in this article suggests that most of the reported genotypes were made available through master’s and doctoral theses that included patients from these centers. We believe that the number of genotyped patients will increase as genetic testing methods become more accessible in the country.

According to the Brazilian Ministry of Health, the coverage of the public neonatal screening program is 82.7% [12]; however, many newborns are screened by the private health sector, so the coverage is probably even higher. There is also evidence of inadequate coverage in poor and isolated communities, such as those in the Amazon (North region) [10]. Furthermore, the Brazilian population has been predominantly concentrated in coastal regions. Currently, 83.5% of Brazilians reside in the Northeast (55.9 million), Southeast (87.3 million), and South (30.6 million) regions [49]. Our data included patients from 16/27 Brazilian states (including the Federal District). The unrepresented states were from the North (*n* = 6/7 states), Central-West (*n* = 2/4), Northeast (*n* = 2/9) and Southeast regions (*n* = 1/4); all 3 states from the South were represented. At least 77.9% of genotyped patients originating from Northeast/Southeast/South regions (*n* = 578). This proportion may be underestimated, as geographic information was unavailable for 16.3% of cases (*n* = 121). Therefore, our findings reflect a predominance of individuals from the most populous areas in the country (South, Southeast and Northeast) and a lower representation of Amazonian (e.g., the North) and non-coastal regions (e.g., the Central-West). Not surprisingly, the genetic findings of our sample reflect those ones found in Europeans countries like Portugal and Spain.

## Conclusions

This study represents the first nationwide effort to compile both published and unpublished PAH genotype data from Brazilian patients with PKU. The results underscore both the predominance of European-derived alleles, the substantial heterogeneity that characterizes a highly admixed population, and provide an overview of the distribution of pathogenic PAH variants across the country. The marked phenotypic variability observed among recurrent genotypes reinforces the importance of standardized phenotyping. Finally, the predominance of cases from more populous coastal regions and the relative underrepresentation of Amazonian and inland areas reveals persistent geographic and socioeconomic disparities in genetic testing and newborn screening coverage, emphasizing the need to expand diagnostic infrastructure and integrate genotyping into routine PKU care to ensure more equitable management across Brazil. These findings may support the development of optimized, targeted sequencing strategies, such as Sanger sequencing, in settings where comprehensive gene analysis is not feasible.

## Supplementary Information

Below is the link to the electronic supplementary material.

Supplementary Material 1

## Data Availability

Supplementary data that are not available in this manuscript or supplementary materials will be shared upon request.

## References

[1] Anikster Y, Haack TB, Vilboux T, Pode-Shakked B, Thöny B, Shen N, et al. Biallelic Mutations in DNAJC12 Cause Hyperphenylalaninemia, Dystonia, and Intellectual Disability. Am J Hum Genet. 2017;100:257–66. 10.1016/j.ajhg.2017.01.002. 28132689 10.1016/j.ajhg.2017.01.002 PMC5294665

[2] Li Y, Tan Z, Zhang Y, Zhang Z, Hu Q, Liang K, et al. A noncoding RNA modulator potentiates phenylalanine metabolism in mice. Science. 2021;373:662–73. 10.1126/science.aba4991. 34353949 10.1126/science.aba4991 PMC9714245

[3] Stelzer G, Rosen N, Plaschkes I, Zimmerman S, Twik M, Fishilevich S, et al. Curr Protocols Bioinf. 2016;54(130). 10.1002/cpbi.5. The GeneCards Suite: From Gene Data Mining to Disease Genome Sequence Analyses. 10.1002/cpbi.5 27322403

[4] Stenson PD, Mort M, Ball EV, Evans K, Hayden M, Heywood S, et al. Hum Genet Springer Berlin Heidelberg. 2017;136:665–77. 10.1007/s00439-017-1779-6. The Human Gene Mutation Database: towards a comprehensive repository of inherited mutation data for medical research, genetic diagnosis and next-generation sequencing studies. 10.1007/s00439-017-1779-6 PMC5429360 28349240

[5] van Spronsen FJ, Blau N, Harding C, Burlina A, Longo N, Bosch AM, Phenylketonuria. Nat Reviews Disease Primers. 2021;7:1–19. 10.1038/s41572-021-00267-0. 10.1038/s41572-021-00267-0 PMC8591558 34017006

[6] Hillert A, Anikster Y, Belanger-Quintana A, Burlina A, Burton BK, Carducci C, et al. The Genetic Landscape and Epidemiology of Phenylketonuria. Am J Hum Genet. 2020;107:234–50. 10.1016/j.ajhg.2020.06.006. 32668217 10.1016/j.ajhg.2020.06.006 PMC7413859

[7] Borrajo GJC. Newborn screening in Latin America at the beginning of the 21st century. J Inher Metab Disea. 2007;30:466–81. 10.1007/s10545-007-0669-9. 10.1007/s10545-007-0669-9 17701285

[8] Chiesa A, Spécola N, Poubel M, Vela-Amieva M, Jurecki E, Vilela DR, et al. Adherence to PKU guidelines among patients with phenylketonuria: A cross-sectional national multicenter survey-based study in Argentina, Brazil, and Mexico. Mol Genet Metabolism Rep. 2024;38:101026. 10.1016/j.ymgmr.2023.101026. 10.1016/j.ymgmr.2023.101026 PMC10700621 38077955

[9] Vieira Neto E, Maia Filho HS, Monteiro CB, Carvalho LM, Tonon T, Vanz AP, et al. Quality of life and adherence to treatment in early-treated Brazilian phenylketonuria pediatric patients. Braz J Med Biol Res. 2018;51:e6709. 10.1590/1414-431X20176709. 10.1590/1414-431X20176709 PMC5731329 29267500

[10] De Souza CFM, Tonon T, Silva TO, Bachega TASS. Newborn screening in Brazil: realities and challenges. J Community Genet [Internet]. 2025. 10.1007/s12687-024-00762-3. [cited 2025 Jan 15]. 39792349 10.1007/s12687-024-00762-3 PMC12321699

[11] Vargas PR, Poubel M, Martins B, Velez P, Vilela D, Mesojedovas D, et al. Patient journey and disease burden characterization of the population with phenylketonuria (PKU) in Brazil: a retrospective analysis through data reported in the public health system administrative database (DATASUS). Lancet Reg Health - Americas. 2025;47:101134. 10.1016/j.lana.2025.101134. 40529850 10.1016/j.lana.2025.101134 PMC12173015

[12] BRASIL. Ministério da Saúde. Secretaria de Atenção Especializada à Saúde. Painel Programa Nacional de Triagem Neonatal (PNTN) - Cobertura Percentual de 2012 a 2024 [Internet]. Portal Gov.br. Triagem Neonatal; 2025 [cited 2026 May 15]. https://www.gov.br/saude/pt-br/composicao/saes/triagem-neonatal/painel. Accessed 15 May 2026.

[13] Garbade SF, Shen N, Himmelreich N, Haas D, Trefz FK, Hoffmann GF, et al. Allelic phenotype values: a model for genotype-based phenotype prediction in phenylketonuria. Genet Med Springer US. 2019;21:580–90. 10.1038/s41436-018-0081-x. 10.1038/s41436-018-0081-x 29997390

[14] van Spronsen FJ, van Wegberg AM, Ahring K, Bélanger-Quintana A, Blau N, Bosch AM, et al. Key European guidelines for the diagnosis and management of patients with phenylketonuria. Lancet Diabetes Endocrinol. 2017;8587:1–14. 10.1016/S2213-8587(16)30320-5. 10.1016/S2213-8587(16)30320-5 28082082

[15] Karczewski KJ, Francioli LC, Tiao G, Cummings BB, Alföldi J, Wang Q, et al. The mutational constraint spectrum quantified from variation in 141,456 humans. Nature. 2020;581:434–43. 10.1038/s41586-020-2308-7. 32461654 10.1038/s41586-020-2308-7 PMC7334197

[16] Naslavsky MS, Scliar MO, Yamamoto GL, Wang JYT, Zverinova S, Karp T, et al. Whole-genome sequencing of 1,171 elderly admixed individuals from Brazil. Nat Commun. 2022;13:1004. 10.1038/s41467-022-28648-3. 35246524 10.1038/s41467-022-28648-3 PMC8897431

[17] BRASIL. Instituto Brasileiro de Geografia e Estatística (IBGE). Resolução n^o^ 1, de 27 de agosto de 2025. Divulga as estimativas de população para Estados e Municípios com data de referência em 1^o^ de julho de 2025 [Internet]. Diário Oficial da União Aug 28, 2025 p. 53. https://ftp.ibge.gov.br/Estimativas_de_Populacao/Estimativas_2025/estimativa_dou_2025.pdf. Accessed 15 May 2026.

[18] Hart RK, Fokkema IFAC, DiStefano M, Hastings R, Laros JFJ, Taylor R, et al. HGVS Nomenclature 2024: improvements to community engagement, usability, and computability. Genome Med. 2024;16:149. 10.1186/s13073-024-01421-5. 39702242 10.1186/s13073-024-01421-5 PMC11660784

[19] Acosta AX, Silva WA, Carvalho TM, Gomes M, Zago MA. Mutations of the phenylalanine hydroxylase (PAH) gene in Brazilian patients with phenylketonuria. Human Mutation. Springer International Publishing; 2001 10.1002/1098-1004(200102)17:2<122::AID-HUMU4>3.0.CO;2-C 11180595

[20] Acosta AX. Análise molecular do gene da fenilalanina hidroxilase em pacientes com fenilcetonúria [Thesis] [Doutorado Direto em Clínica Médica]. [Ribeirão Preto]: Universidade de São Paulo; 2001. 10.11606/T.17.2001.tde-18032024-125357

[21] Steiner CE, Acosta AX, Guerreiro MM, Marques-de-Faria AP. Genotype and natural history in unrelated individual with phenylketonuria and autistic behavior. Arq Neuro-Psiquiatr. 2007;65:202–5. 10.1590/S0004-282X2007000200003. 10.1590/s0004-282x2007000200003 17607414

[22] Santos LL dos. Frequência das mutações I65T, Y414C, R252W e R261Q em indivíduos com fenilcetonúria do estado de Minas Gerais. [Thesis]: UNIVERSIDADE FEDERAL DE MINAS GERAIS; 2004.

[23] Magalhães, M de C. Frequencia das mutações v388m, r408w, ivs10nt11 e ivs12nt1 em indivíduos com fenilcetonúria do estado de minas gerais.[Thesis] UNIVERSIDADE FEDERAL DE MINAS GERAIS (UFMG). 2003

[24] Santos LL dos, Magalhães MDC, Reis ADO, Starling ALP, Januário JN, Fonseca CG da, et al. Frequencies of phenylalanine hydroxylase mutations I65T, R252W, R261Q, R261X, IVS10nt11, V388M, R408W, Y414C, and IVS12nt1 in Minas Gerais, Brazil. Genetics and molecular research : GMR. 2006;5:16–23. 16755493

[25] Santos LL, Fonseca CG, Starling ALP, Januário JN, Aguiar MJB, Peixoto MGCD, et al. Variations in genotype-phenotype correlations in phenylketonuria patients. Genet Mol Res. 2010;9:1–8. 10.4238/vol9-1gmr670. 20082265 10.4238/vol9-1gmr670

[26] Santos, LL dos. AS BASES MOLECULARES DA FENILCETONÚRIA NO ESTADO DE MINAS GERAIS. [Thesis] UNIVERSIDADE FEDERAL DE MINAS GERAIS; 2007.

[27] Lourenço Pollice E. Caracterização molecular da fenilcetonuria em pacientes da região de Campinas [Internet] [Doutor em Ciências Médicas]. [Campinas, SP]: Universidade Estadual de Campinas; 2008 [cited 2025 Apr 25]. 10.47749/T/UNICAMP.2008.431843

[28] Vieira Neto E, Laranjeira F, Quelhas D, Ribeiro I, Seabra A, Mineiro N, et al. Genotype-phenotype correlations and BH 4 estimated responsiveness in patients with phenylketonuria from Rio de Janeiro, Southeast Brazil. Mol Genet Genomic Med. 2019;7:1–16. 10.1002/mgg3.610. 10.1002/mgg3.610 PMC6503030 30829006

[29] Vieira Neto E. Fenilcetonúria no Rio de Janeiro: perfil mutacional e desfechos do tratamento precoce. [Thesis]: UNIVERSIDADE FEDERAL DO RIO DE JANEIRO; 2018.

[30] Vieira Neto E, Laranjeira F, Quelhas D, Ribeiro I, Seabra A, Mineiro N, et al. Mutation analysis of the PAH gene in phenylketonuria patients from Rio de Janeiro, Southeast Brazil. Molecular Genetics and Genomic Medicine. 2018;6:575–91. 10.1002/mgg3.408. 10.1002/mgg3.408 PMC6081236 29749107

[31] Amorim Boa Sorte TRS. Estudo de bases moleculares de Fenilcetonúria no Nordeste do Brasil [Internet] [Thesis]. 2010 [cited 2025 Apr 25]. https://www.arca.fiocruz.br/handle/icict/4310. Accessed 25 Apr 2025.

[32] Santana Santos E de, Rocha MAA, Costa D, Amorim T, Acosta AX. Caracterização genético-clínica de pacientes com fenilcetonúria no Estado de Alagoas. Sci Med. 2012;22:64–70.

[33] Silva CAN. Caracterização clínica e molecular de pacientes com fenilcetonúria no Estado da Bahia [Internet]. 2018 [cited 2025 Apr 25]. https://www.arca.fiocruz.br/handle/icict/30493. Accessed 25 Apr 2025.

[34] Bonfim-Freitas PE. Identificação e caracterização molecular de mutações causadoras de fenilcetonúria em pacientes do estado do Pará. [Thesis]: UNIVERSIDADE FEDERAL DO PARÁ; 2006.

[35] Bonfim-Freitas PE, Andrade RS, Ribeiro‐dos‐Santos ÂK, Silva LCS. Molecular characterization of phenylketonuria patients from the North Region of Brazil: State of Pará. Molec Gen Gen Med. 2023;11:e2224. 10.1002/mgg3.2224. 10.1002/mgg3.2224 PMC10568386 37421234

[36] Costa RD. Estudo das mutações IVS10NT-11G>A, V388M, R261Q, R261X, R252W E R408W no gene da fenilalanina hidroxilase em pacientes com fenilcetonúria do estado de Mato Grosso. [Thesis] Universidade Federal de Mato Grosso; 2017.

[37] Costa RD, Galera BB, Rezende BC, Venâncio AC, Galera MF. Identification of mutations in the PAH gene in PKU patients in the state of Mato Grosso. Revista Paulista de Pediatria. 2020;38. 10.1590/1984-0462/2020/38/2018351. 10.1590/1984-0462/2020/38/2018351 PMC7025444 32074228

[38] Santana Da Silva LC. Identificação e caracterização molecular de mutações e polimorfismos no gene da fenilalanina hidroxilase em fenilcetonúricos do sul do país. 2000 [Thesis] UNIVERSIDADE FEDERAL DO RIO GRANDE DO SUL.

[39] Santana Da Silva LC, Santos Carvalho T, Britto Da Silva F, Morari L, Aguirres Fachel Â, Pires R, et al. Molecular characterization of phenylketonuria in South Brazil. Mol Genet Metab. 2003;79:17–24. 10.1016/S1096-7192(03)00032-5. 12765842 10.1016/s1096-7192(03)00032-5

[40] Tresbach RH, Sperb-Ludwig F, Ligabue-Braun R, Tonon T, de Oliveira Cardoso MT, Heredia RS, et al. Phenylketonuria Diagnosis by Massive Parallel Sequencing and Genotype-Phenotype Association in Brazilian Patients. Genes. 2020;12:20. 10.3390/genes12010020. 33375644 10.3390/genes12010020 PMC7824641

[41] Tresbach RH. Análise do genótipo e fenótipo em pacientes brasileiros com hiperfenilalaninemias e doença da urina do xarope do bordo. [Thesis] Universidade Federal do Rio Grande do Sul. 2021.

[42] Nunes AJ, Franceschetto BF, Da Gama L, Poloni S, Refosco LF, Tonon T, et al. The Influence of Phenylalanine Fluctuations and Intake on a 24 h Sapropterin Responsiveness Test in Patients with Phenylketonuria. Children. 2025;12:541. 10.3390/children12050541. 40426720 10.3390/children12050541 PMC12110409

[43] de Souza AM, Resende SS, de Sousa TN, de Brito CFA. A systematic scoping review of the genetic ancestry of the brazilian population. Genet Mol Biology. 2019;42:495–508. 10.1590/1678-4685-gmb-2018-0076. 10.1590/1678-4685-GMB-2018-0076 PMC6905439 31188926

[44] Ferreira F, Azevedo L, Neiva R, Sousa C, Fonseca H, Marcão A, et al. Phenylketonuria in Portugal: Genotype–phenotype correlations using molecular, biochemical, and haplotypic analyses. Mol Genet Genomic Med. 2021;9:1–12. 10.1002/mgg3.1559. 10.1002/mgg3.1559 PMC8104178 33465300

[45] Aguirre AS, Haro E, Campodónico A, Arias-Almeida B, Mendoza A, Pozo-Palacios J, et al. Expanding diversity within phenylketonuria in ecuadorian patients: genetic analysis and literature review of newborn screenings. BMC Pediatr. 2024;24:739. 10.1186/s12887-024-05140-z. 39548419 10.1186/s12887-024-05140-z PMC11566563

[46] Blau N, Hennermann JB, Langenbeck U, Lichter-Konecki U. Diagnosis, classification, and genetics of phenylketonuria and tetrahydrobiopterin (BH4) deficiencies. Mol Genet Metab. 2011;104:2–9. 10.1016/j.ymgme.2011.08.017. 10.1016/j.ymgme.2011.08.017 21937252

[47] Heintz C, Cotton RGH, Blau N. Tetrahydrobiopterin, its Mode of Action on Phenylalanine Hydroxylase, and Importance of Genotypes for Pharmacological Therapy of Phenylketonuria. Hum Mutat. 2013;34:927–36. 10.1002/humu.22320. 23559577 10.1002/humu.22320

[48] Sacramento AP, Almeida AO, Portugal CM, Losco LN, de Souza AB, Galvão L et al. Coordenação de incorporação de tecnologias – CITEC/CGGTS/DGITIS/SCTIE/MS. Report No: 253.

[49] IBGE IB de G e E. Censo Demográfico 2022 [Internet]. 2022. https://www.ibge.gov.br/estatisticas/sociais/populacao/22827-censo-demografico-2022.html?edicao=35938

